# Anti-Angiogenic Agents in Management of Sarcoma Patients: Overview of Published Trials

**DOI:** 10.3389/fonc.2020.594445

**Published:** 2020-11-24

**Authors:** Pierre-Yves Cren, Loïc Lebellec, Thomas Ryckewaert, Nicolas Penel

**Affiliations:** ^1^ Lille University, Medical School, Lille, France; ^2^ Medical Oncology Unit, Tourcoing Hospital, Tourcoing, France; ^3^ Medical Oncology Department, Centre Oscar Lambret, Lille, France

**Keywords:** Choi criteria, clinical trial, multikinase inhibitor, non-adipocytic soft tissue sarcoma, sarcoma

## Abstract

We reviewed all fully published clinical trials assessing anti-angiogenic agents in sarcoma patients (last issue, January 13, 2020). Anti-angiogenic macromolecules (e.g., bevacizumab or ombrabulin) provide disappointing results. Many multikinase inhibitors have been assessed with non-randomized phase II trials with limited samples and without stratification according to histological subtypes, therefore interpretation of such trials is very challenging. On the contrary, pazopanib, regorafenib, and sorafenib have been assessed using double-blind placebo-controlled randomized phase II or phase III trials. Compared to placebo, sorafenib demonstrates activity in desmoid-type fibromatosis patients. Based on results of phase 3 trial, pazopanib had obtained approval for treatment of pretreated non-adipocytic soft tissue sarcoma. Regorafenib is currently assessed in several clinical settings and provides significant improvement of progression-free survival in pre-treated non-adipocytic soft tissue sarcoma and in advanced pretreated osteosarcoma. Multikinase inhibitors are a breakthrough in sarcoma management. Many trials are ongoing. Nevertheless, predictive factors are still missing.

**Graphical Abstract:**
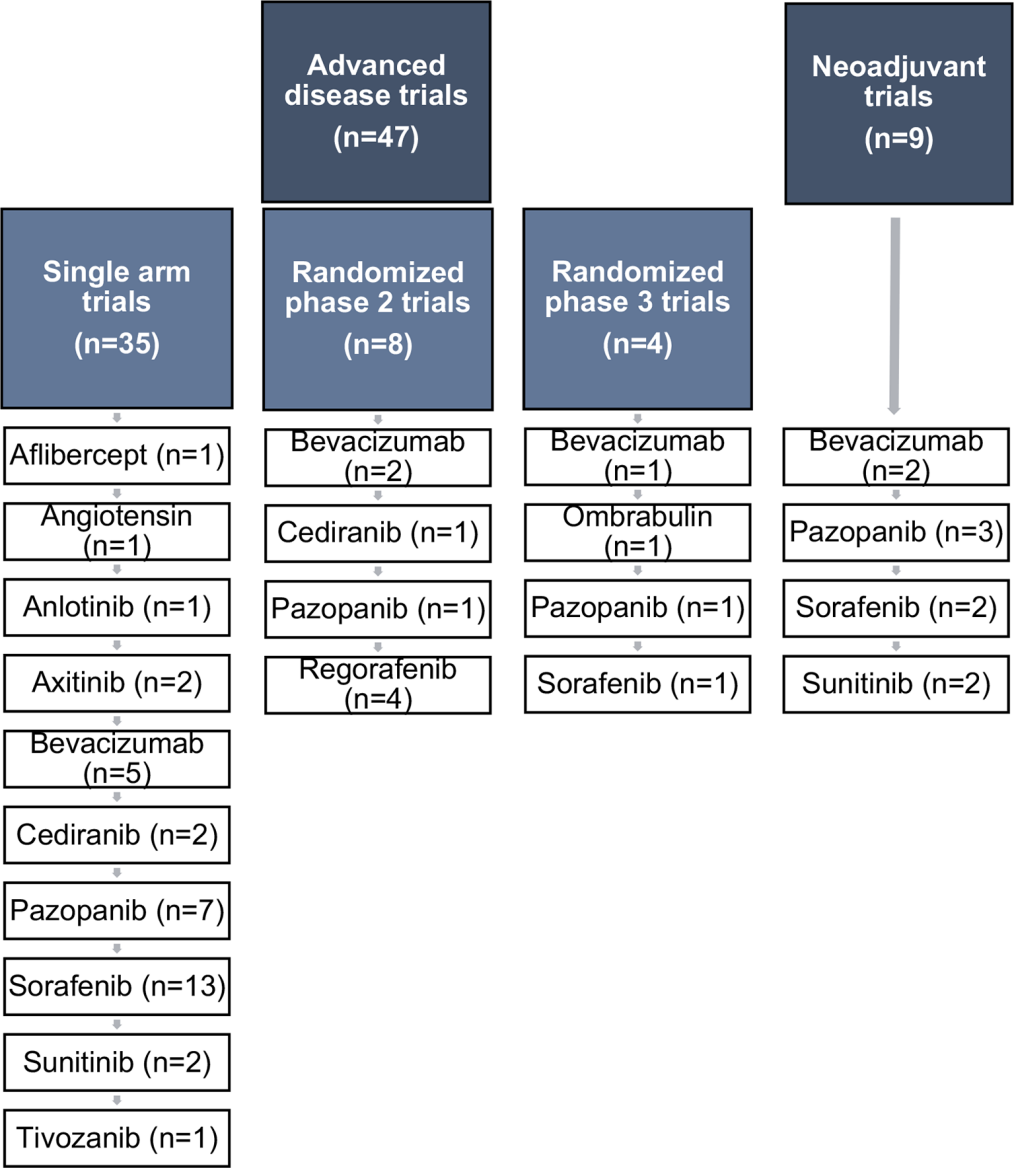
We systematically reviewed all fully published clinical trials assessing anti-angiogenic agents in sarcoma patients, using Medline (last issue 13 January 2020): n = 56.

## Introduction

Sarcoma represents less than 2% of adult malignancies and about 15% to 20% of malignancies in children and adolescents/young adults. Sarcomas account for more than 80 different clinico-pathological entities with different clinical behavior. Systemic treatments are used as (neo)adjuvant treatment for curative-intent management of localized osteosarcoma and Ewing sarcoma, and as palliative treatment in advanced soft tissue sarcoma (1–3). The role of (neo)adjuvant treatment in localized soft tissue sarcoma remains debated. Because of heterogeneity of sarcomas, recommended systemic treatments widely differed according to histological subtypes: kinase inhibitor targeting c-Kit and PDGFR-α in gastro-intestinal stromal tumors, hormonal therapy in some particular rare entities, molecular targeted therapies in some rare entities, and chemotherapy in most of clinical settings. In the past two decades, many clinical trials assessed the therapeutic role of checkpoint inhibitors in sarcoma patients, but results remain disappointing. On the contrary, there is a growing body of evidence that anti-angiogenic, and especially multikinase inhibitors constitute a breakthrough in management of sarcoma patients. The objective of the present study was to summarized the published data about activity of antiangiogenic agents in sarcoma patients.

## Methods

**Figure 1:**
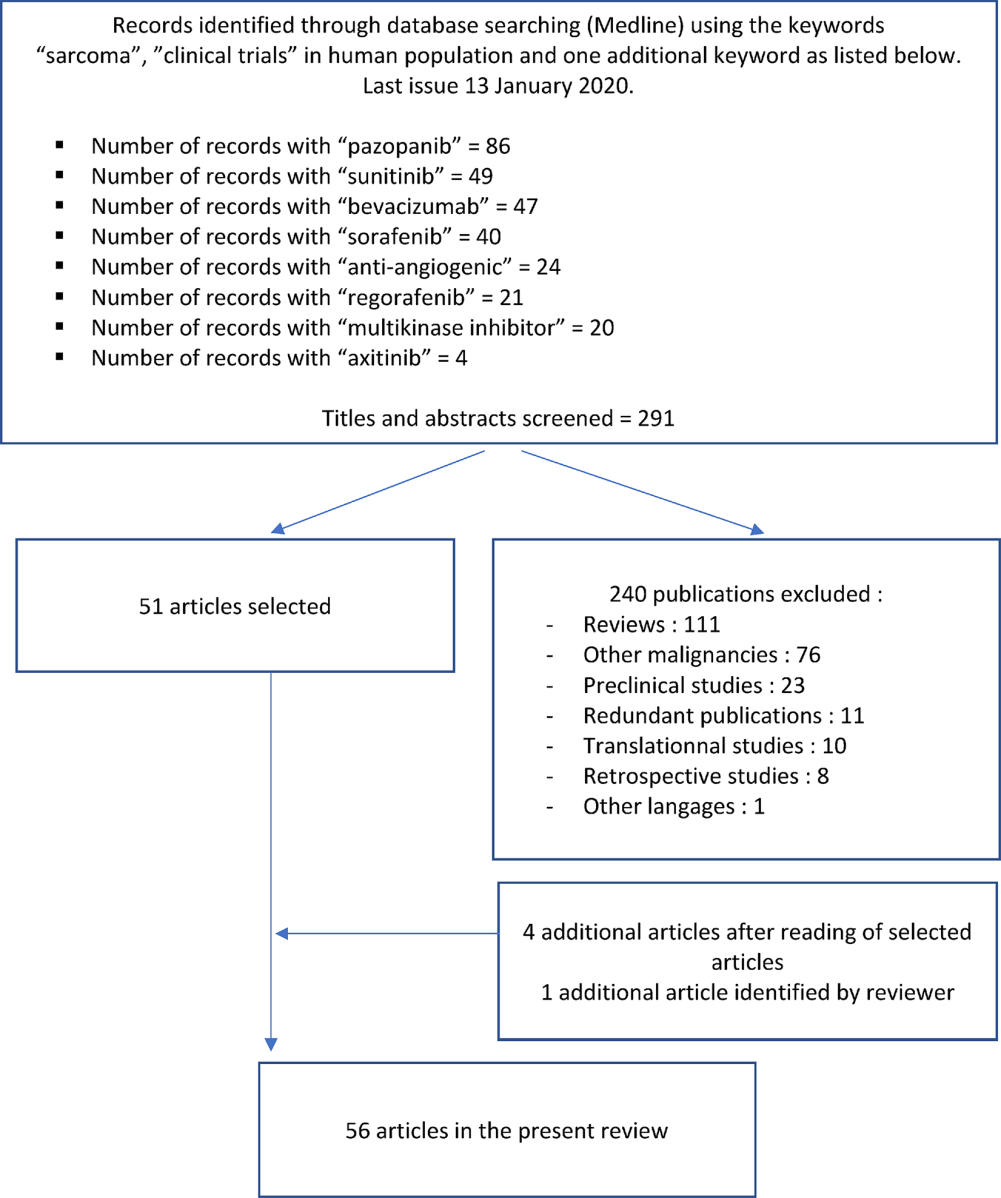
Search strategy.

In the present review, we have summarized published results of clinical trials assessing the role of anti-angiogenic agents in sarcoma patients. This research used Medline (last issue January 13, 2020). We have searched prospective studies (phase I, phase II, and phase III) trials assessing the activity of anti-angiogenic (alone or in combination) for management of sarcoma patients (adults and children). The following keywords have been systematically used: “sarcoma” and “clinical trials”, with the following terms: “pazopanib” (86 abstracts), “sunitinib” (49 abstracts), “bevacizumab” (47 abstracts), “sorafenib” (40 abstracts), “anti-angiogenic” (24 abstracts), “regorafenib” (21 abstracts), “multikinase inhibitors” (20 abstracts) and “axitinib” (4 abstracts). Articles have been selected after reading of these 291 abstracts. We excluded 240 publications. Finally, we added four additional references after reading of selected articles and one additional article identified by reviewer. A total of 56 articles are therefore included in this review (**Figure 1**). We have excluded data confusing on gastro-intestinal stromal tumors or so-called “carcinosarcoma.”

In every phase II and III trials, we have collected the following pieces of information: number of cases, documented disease progression required at study entry, histological subtypes, investigational treatment, objective response rate, median progression-free survival, 3 and 6 months progression-free survival, median overall survival.

Anti-angiogenic agents and their inherent mechanism of actions are listed in **Table 1**. For each kinase inhibitors, we have listed the targets and the maximal inhibitory concentration 50 (IC50).

**Table 1:** Anti-angiogenic agents and their mechanism of action.

	Multi-kinase inhibitors (targets and IC-50 in nM)										
	VEGFR-1	VEGFR-2	VEGFR-3	PDGFRα	PDGFRβ	c-Kit	RET	RAF	FLT3	FGFR-1
Anlotinib (4)	26.9	0.2	0.7	–	115.0	14.8	–	–	6.4	11.7
Axitinib (5)	0.1	0.2	0.1–0.3	5.0	1.6	1.7	>1,000	–	>1,000	–
Cediranib (6)	1.2	–	–	36.0	5.0	2.0	–	–	5.0	–
Pazopanib (7)	10.0	30.0	47.0	71.0	84.0	74.0	>1,000	–	>1,000	80.0
Regorafenib (8)	13.0	4.2	46.0		22.0	7.0	1.5	2.5	–	
Sorafenib	–	90.0	20.0	50.0–60.0	50.0–60.0	68.0	100.0–150.0	5.0–10.0	46.0	64.0
Sunitinib	10.0	10.0	10.0	5.0–10.0	10.0	13.0	100–200	–	1–10	437.0
Tivozanib (9)	30.0	6.5	15.0	40.0	49.0	78.0	–	–	–	530.0
**Other agents**											
Aflibercept			It is a recombinant fusion protein that traps VEGF-A, VEGF-B and PlGF								
Angiotensin			It is a protein that regulates vasoconstriction and blood pressure.								
Bevacizumab			It is a recombinant humanized monoclonal antibody that bocks VEGF-A.								
Ombrabulin			It is a synthetic analogue of combrestatin A4 that acts as vascular-disrupting agent since it binds the colchicine binding site of endothelial cell tubulin and then induce apoptosis of endothelial cells and blood vessels collapse.								

## Results

### Synthesis Drug by Drug

Literature data consists in single-arm trials (**Table 2**), randomized phase 2 trials (**Table 3**) and phase 3 trials (**Table 4**). Interpretation of randomized trials appears more straightforward and convincing since there is an internal comparator and the results of both arms could weight whatever the trial is a comparative (phase 3 trials and some phase 2 trials) or a non-comparative trial (most of phase 2 trials). On the contrary, the interpretation of single-arm trial is much more challenging, the data depend on the true activity of the investigational drug but also on the tumor/patient selection (histological subtypes that could include indolent diseases or very aggressive diseases, tumor grade, metastatic burden, intolerance to *versus* failure of prior line …). Furthermore, single-arm trials, median progression-free survival (PFS) and progression-free survival rates (PFR) at fixed time point must be interpreted with caution since depending on the time interval between two tumor assessments. When summarized literature data, two methodological points had to be stressed: (i) evidence of disease progression at study entry, and (ii) the number of prior treatments. There is a minority of non-randomized trials requiring evidence of disease progression at study entry (14, 20, 23, 24, 27). The description of prior treatment exposure is incomplete in most published trials. Nevertheless, to measure and to discuss the therapeutic role of the anti-angiogenic agents, we refer to the criteria of the EORTC-STBSG that defined a promising drug in phase II trials, as a drug providing in patients with pretreated soft tissue and visceral sarcoma a PFR-3 (progression-free rate at 3 months) ≥40% and PFR-6 (progression-free rate at 6 months) ≥14% (53). Similarly, promising drug is defined as drug providing PFR-6 ≥30% in first line. To the best of our knowledge there is no similar criteria and threshold for bone sarcoma patients. Furthermore, occurrence of confirmed objective response is a convincing evidence for pharmacodynamic activity of the drug.

**Table 2:** Single-arm trials in sarcoma patients.

Reference	Drug	Primaries	Histological subtypes	e-PD	n	Chemo naive, n (%)	1 prior line, n (%)	ORR	PFR-3	PFR-6	Median PFS (months)	Median OS (months)
**Mackay et al. Gynecol Oncol 2012** (10)	aflibercept	Uterine (advanced)	Leiomyosarcoma	NM	41	16 (39%)	18 (44%)	0	?	17%	?	18.1
**Savage et al. Sarcoma 2016** (11)	angiotensin	All (advanced)	all	NM	20	0	?	0	45%	?	2.7	10.2
**Chi et al. CCR 2018** (12)	Anlotinib	Soft tissue (advanced)	ASPS	NM	13	?	?	6 (46%)	77%	77%	21.0	Not reached
**Chi et al. CCR 2018** (12)	Anlotinib	Sott tissue (advanced)	Clear cell sarcoma	NM	7	?	?	1 (14%)	54%	54%	11.0	16.00
**Chi et al. CCR 2018** (12)	Anlotinib	Soft tissue (advanced)	Fibrosarcoma	NM	18	?	?	2 (11%)	81%	44%	5.6	12.0
**Chi et al. CCR 2018** (12)	Anlotinib	Soft tissue (advanced)	Leiomyosarcoma	NM	26	?	?	2 (8%)	75%	69%	11.0	15.0
**Chi et al. CCR 2018** (12)	Anlotinib	Soft tissue (advanced)	Liposarcoma	NM	13	?	?	1 (8%)	63%	53%	5.6	13.0
**Chi et al. CCR 2018** (12)	Anlotinib	Soft tissue (advanced)	Other histologies	NM	23	?	?	0 (0%)	44%	24%	2.8	8.8
**Chi et al. CCR 2018** (12)	Anlotinib	Soft tissue (advanced)	Synovial sarcoma	NM	47	?	?	8 (17%)	75%	53%	7.7	12.0
**Stachiotti et al. EJC 2019** (13)	Axitinib	Soft tissue (advanced)	SFT	Yes	17	9 (53%)	?	1/9 (11%)	?	?	9.4	25.3
**Wilky et al. Lancet Oncol 2019** (14)	Axitinib + Pembrolizumab	Soft tissue (advanced)	ASPS	Yes	12	?	?	6/11 (55%)	72%	38%	12.4	Not reached
**Wilky et al. Lancet Oncol 2019** (14)	Axitinib + Pembrolizumab	Soft tissue (advanced)	Non-ASPS	Yes	21	?	?	2/21 (10%)	62%	?	3.0	13.1
**Agulnik et al. Ann Oncol 2013** (15)	Bevacizumab	Soft tissue (advanced)	angiosarcoma	NM	23	?	?	2/23 (9%)	?	?	3	13.2
**Agulnik et al. Ann Oncol 2013** (15)	Bevacizumab	Soft tissue (advanced)	EHE	NM	7	?	?	2/7 (29%)	?	?	9.8	35.5
**D’Amado et al. JCO 2005 **(16 ****)	Doxorubicin + bevacizumab	Soft tissue (advanced)	Leiomyosarcoma	NM	17	11 (65%)	6 (35%)	2 (12%)	?	?	?	16.0
**Dickson et al. Sarcoma 2015** (17)	Bevacizumab + Gemcitabine + Docetaxel	Soft tissue (advanced)	All	NM	35	29 (83%)		17/35 (49%)	76%	65%	7.5	28.8
**Monga et al. Cancers 2018** (18)	Bevacizumab + Gemcitabine + Docetaxel + Valproic Acid	Soft tissue (advanced)	All	?	46	12 (26%)	15 (33%)	7/41 (17%)			5.7	12.9
**Verschraegen et al. Ann Oncol 2012** (19)	Bevacizumab + Gemcitabine + Docetaxel	Soft tissue (neoadjuvant)	All	NM	15	15 (100%)	0	6/15 (40%)	?	?	?	2y-OS rate : 69%
**Verschraegen et al. Ann Oncol 2012** (19)	Bevacizumab + Gemcitabine + Docetaxel	Soft tissue (advanced)	All	NM	20	20 (100%)	0	5/20 (25%)	?	?	5.0	11.0
**Kim et al. Oncologist 2019** (7)	Pazopanib	Soft tissue (advanced)	ASPS	NM	6	4 (67%)	1 (17%)	1/6 (17%)	100%	50%	5.5	Not reached
**Martin-Broto et al. Lancet Oncol 2019** (20)	Pazopanib	Soft tissue (advanced)	STF (malignant or dedifferentiated)	Yes	36	24 (67%)	3 (8%)	2/35 (6%)			5.6	Not reached
**Pautier et al. EJC 2020** (21)	Pazopanib + gemcitabine	Uterine and soft tissue (advanced)	Leiomyosarcoma	NM	105	0 (0%)	105 (100%)	24/105 (23%)			6.5	24.3
**Samuels et al. Cancer 2017** (22)	Pazopanib	Soft tissue (advanced)	Liposarcoma (intermediate or high-grade)	NM	41	7 (17%)	10 (24%)	1/41 (2%)	68%	39%	4.4	12.6
**Sleijfer JCO 2009** (23)	Pazopanib	Soft tissue (advanced)	Liposarcoma	Yes	19	1 (5%)		0 (0%)	26%		2.6	6.6
**Sleijfer JCO 2009** (23)	Pazopanib	Soft tissue (advanced)	Leiomyosarcoma	Yes	42	1 (2%)		1 (2%)	44%		3.0	11.8
**Sleijfer JCO 2009** (23)	Pazopanib	Soft tissue (advanced)	Synovialosarcoma	Yes	38	0 (0%)		5 (13%)	49%		5.3	10.3
**Sleijfer JCO 2009** (23)	Pazopanib	Soft tissue (advanced)	Other histologies	Yes	43	0 (0%)		3 (7%)	39%		3.0	9.9
**Stacchiotti Lancet Oncol 2019** (24)	Pazopanib	Soft tissue (advanced)	extraskeletal myxoid chondrosarcoma	Yes	26	21 (81%)	2 (8%)	4/26 (15%)			19.0	Not reached
**Subbiah CCR 2017** (25)	Pazopanib + trametinib	Soft tissue (advanced)	All	NM	25	0 (0%)		2/25 (8%)			2.3	9.0
**Bompas Anna Oncol 2015** (26)	Sorafenib	Bone (advanced)	Chordoma	NM	27	15 (56%)	?	2/27 (7%)		85%	Not reached	Not reached
**Chevreau et al. Cancer 2013** (27)	Sorafenib	Soft tissue	EHE	Yes	15	10 (67%)	?	2/15 (13%)		38%	6.0	Not reached
**D’Adamo et al. The Oncologist 2019** (28)	Sorafenib + DTIC	Soft tissue (advanced)	LMS+MPNST+SS	NM	37	16 (43%)	11 (30%)	5/37 (14%)	51%	25%	3.3	13.2
**Garcia Del Muro Invest New Dugs 2018** (29)	Sorafenib + Ifosfamide	Soft tissue (advanced)	All	NM	35	3 (9%)		6/35 (17%)	66%	37%	4.8	16.2
**Grignani et al. Ann Oncol 2012** (30)	Sorafenib	Bone (advanced)	Osteosarcoma	NM	35	0 (0%)		3/35 (9%)		17%	4.0	7.0
**Grignani et al. Lancet Oncol 2015 (** 30 **)**	Sorafenib + everolimus	Bone (advanced)	Osteosarcoma	NM	38	0 (0%)		2/38 (5%)		45%	5	11
**Von Mehren et al. Cancer 2012** (31)	Sorafenib	Soft tissue (advanced)	All	NM	37	18 (49%)		0/37 (0%)			3.0	17.0
**Pacey et al. Invest New Drugs 2011** (32)	Sorafenib	Soft tissue (advanced)	All	NM	21	0 (0%)	7 (33%)	3/21 (14%)	14%	0%		
**Raut et al. PLOSOne 2012** (33)	Sorafenib	Soft tissue (advanced)	All	NM	15	1 (7%)		0/15 (0%)				
**Ray-Coquard et al. Oncologist 2012** (34)	Sorafenib	Soft tissue (advanced)	angiosarcoma	NM	41	11 (27%)	16 (39%)	6/41 (15%)		12%		
**Santoro et al. Ann Oncol** **2013** (35)	Sorafenib	Soft tissue (advanced)	All	NM	101	0 (0%)	40 (40%)	11/76 (14%)		35%	4.2	11.9
**Valentin et al. Invest New Drugs 2013** (36)	Sorafenib	Soft tissue	SFT	NM	5	3 (60%)	2 (40%)	0				19.7
**Gounder et al.** **Clin Cancer res 2011** (37)	Sorafenib	Soft tissue (advanced)	Desmoid tumors	NM	26	11 (42%)		6/24 (25%)				
**George et al. J Clin Oncol 2009** (38)	Sunitinib	Soft tissue (advanced)	All	NM	48			1/48 (2%)		14.5%		
**Jo et al. Invest New Drugs 2014** (39)	Sunitinib	Soft tissue (advanced)	Desmoid tumors	NM	19	11 (58%)	4 (21%)	5 (26%)				
**Agulnik et al. Ann Oncol 2017** (15)	Tivozanib	Soft tissue (advanced)	All	NM	58	0 (0%)	20 (34%)	2/58 (3%)			3.5	12.2
**Cohen et al. Pediatr Blood Cancer 2019** (40)	Cediranib	Soft tissue	ASPS	NM	7	3 (43%)		0/7 (0%)				
**Kummar et al. JCO 2013** (41)	Cediranib	Soft tissue	ASPS	NM	46	18 (39%)	18 (39%)	15/43 (35%)		36/43 (84%)		

e-PD, disease progression at entry in the study; ORR, objective response rate according to RECIST; PFR, progression-free survival rate; PFS: progression-free survival; OS, overall survival; NM, not mandatory; ASPS, alveolar soft tissue part sarcoma; SFT, solitary fibrous tumor; EHE, epithelioid hemangioendothelioma; LMS, leiomyosarcoma; MPNST, malignant peripheral nerve sheath tumor; SS, synovial sarcoma.

**Table 3:** Randomized Phase 2 trials assessing anti-angiogenic agents.

Ref.	Endpoint	Arm A	Arm B	Test
**Ray Coquard et al. JCO** **2015** (42)	Advanced angiosarcoma			
		Weekly paclitaxel (n = 24)	Weekly paclitaxel + bevacizumab (n = 25)	
	Primary: PFS (median; in months)	6.6	6.6	–
	ORR OS (median; in months)	46% 19.5	28% 15.9	- -
**Chisholm et al. EJC** **2017** (43)	Advanced chemo-naïve soft tissue sarcoma in children			
		Chemotherapy (n = 80)	Chemotherapy + bevacizumab (n = 74)	
	Primary: EFS (median; in months)	14.9	20.6	HR = 0.93 (95% CI: 0.61–1.41); p = 0.72
	ORR OS (median; in months)	36% 20.5	54% 25.0	- -
**Judson et al. Lancet Oncol** **2019** (44)	Advanced alveolar soft tissue part sarcoma			
		Placebo (n = 16)	Cediranib (n = 32)	
	Primary: tumor size change	+13·4%	-8·3%	p = 0.0010
	ORR PFS (median; in months) OS (median; in months)	0% 4.9 47.3	19% 10.1 27.8	p = 0.072 HR = 0.82 (90% CI : 0.47–1.43); p = 0.28 p = 0.48 (log-rank)
**Toulmonde et al. Lancet Oncol** **2019** (45)	Progressing desmoid fibromatosis			
		Methotrexate-vinblastine (n = 4)	Pazopanib (n = 48)	
	Primary: PFR-6	45%	84%	–
	ORR	25%	37%	–
**Mir et al. Lancet Oncol** **2016** (46)	Advanced chemotherapy-pretreated liposarcoma			
		Placebo (n = 23)	Regorafenib (n = 20)	
	Primary: PFS (median; in months)	1.7	1.1	HR = 0.89 (95% CI : 0.48–1.64); p = 0.70
	ORR OS (median; in months)	0% 8.8	0% 4.7	- -
**Mir et al. Lancet Oncol** **2016** (46)	Advanced chemotherapy-pretreated leiomyosarcoma			
		Placebo (n = 28)	Regorafenib (n = 28)	
	Primary: PFS (median; in months)	1.8	3.7	HR = 46 (95% CI : 0.26–0.80); p = 0.0045
	ORR OS (median; in months)	4% 9.1	0% 21.0	- -
**Mir et al. Lancet Oncol** **2016** (46)	Advanced chemotherapy-pretreated synovial sarcoma			
		Placebo (n = 14)	Regorafenib (n = 13)	
	Primary: PFS (median; in months)	1.0	5.6	HR = 0.10 (95% CI : 0.03–0.35); p<0.0001
	ORR OS (median; in months)	0% 6.7	8% 13.4	- -
**Mir et al. Lancet Oncol** **2016** (46)	Advanced chemotherapy-pretreated “other” sarcomas			
		Placebo (n = 27)	Regorafenib (n = 28)	
	Primary: PFS (median; in months)	1.0	2.9	HR = 0.46 (95% CI : 0.25–0.82); p = 0.0061
	ORR OS (median; in months)	0% 9.5	11% 12.1	- -
**Penel et al. EJC 2020** (47)	Advanced both chemotherapy and pazopanib pretreated non-adipocytic sarcomas			
		Placebo (n = 18)	Regorafenib (n = 19)	
	Primary: PFS (median; in months)	1.1	2.1	HR = 0.33 (95%-CI: 0.15–0.74); p = 0.007
	ORR OS (median; in months)	0% 8.2	0% 17.8	- HR = 0.49 (95%-CI: 0.23–1.06); p = 0.07
**Duffaud et al. Lancet Oncol** **2019** (48)	Advanced chemotherapy pretreated osteosarcoma			
		Placebo (n = 12)	Regorafenib (n = 26)	
	Primary: PFS (median; in months)	1.0	4.1	–
	ORR OS (median; in months)	0% 5.9	8% 11.3	- -
**Davis et al. JCO** **2019** (49)	Advanced chemotherapy pretreated osteosarcoma			
		Placebo (n = 20)	Regorafenib (n = 22)	
	Primary: PFS (median; in months)	1.7	3.6	HR = 0.42 (95% CI: 0.21–0.85); p = 0.017
	ORR OS (median; in months)	0% 13.4	13.6% 11.1	- HR = 1.26 (95% CI: 0.51–3.13); p = 0.62

ORR, objective response rate according to RECIST; PFR, progression-free survival rate; PFS, progression-free survival; OS, overall survival; HR, hazard ratio; 95% CI, 95% confidence interval.

**Table 4:** Randomized Phase 3 trials assessing anti-angiogenic agents.

Ref.	Endpoint	Arm A	Arm B	Test
**Blay et al.** **Lancet Oncol.** **2015** (50)	Advanced chemotherapy pre-treated soft tissue sarcoma			
		cisplatin (n = 179)	cisplatin + ombrabulin (n = 176)	
	Primary: PFS (median; in months)	1.4	1.5	HR = 0.76 (95% CI, 0.59–0.98); p = 0.0302
	ORR OS (median; in months)	1% 9.3	4% 11.4	- HR = 0.85 (95% CI, 0.67–1.09); p = 0.1970
**Hensley et al.** **JCO** **2015** (51)	Advanced uterine leiomyosarcoma			
		Gemcitabine-docetaxel (n = 54)	Gemcitabine-docetaxel + bevacizumab (n = 53)	
	Primary: PFS (median; in months)	6.2	4.2	HR = 1.12 (95% CI : 0.74–1.70); p = 0.58
	ORR OS (median; in months)	32% 26.9	36% 23.3	- HR = 1.07 (95% CI : 0.63–1.81); p = 0.81
**Van der Graaf et al. Lancet Oncol** **2012** (52)	Advanced chemotherapy-pretreated non-adipocytic soft tissue sarcoma			
		placebo (n = 123)	Pazopanib (n = 246)	
	Primary: PFS (median; in months)	1.6	4.6	HR = 0·31, (95% CI 0·24–0·40); p<0·0001
	ORR OS (median; in months)	0% 10.7	6% 12.5	- HR = 0.86, (95%-CI : 0.67–1.11); p = 0.25
**Gounder et al.** **N Engl J Med** **2018** [40	Sorafenib for advanced and refractory desmoid tumors			
		placebo (n = 37)	Sorafenib (n = 50)	
	Primary: PFS (median; in months)	Not reached	Not reached	HR = 0.13, (95% CI: 0.05 to 0.31); p<0,001
	ORR PFR at 12 months PFR at 24 months	20% 46% 36%	33% 89% 81%	

ORR, objective response rate according to RECIST; PFR, progression-free survival rate; PFS, progression-free survival; OS, overall survival; HR, hazard ratio; 95% CI, 95% confidence interval.

#### Non Tyrosine Kinase Inhibitors

##### Angiotensin

Angiotensin (Ang-(1–7)), a pro-angiogenic peptide regulating vasoconstriction (**Table 1**), provided a PFR-3 of 45% (9/20) in one single-arm phase 2 trial (NCT01553539) in a very heterogeneous group of tumors. Furthermore, there was no reported objective response and median PFS of 2.7 months (11). The short-life of Ang-(1-7) seems to be the key limitation of its therapeutic use. To the best of our knowledge the development of this drug is definitely discontinued.

##### Ombrabulin (AVE8062)

Ombrabulin is a vascular disrupting agent (**Table 1**). Preclinical data suggested synergistic effect with cisplatin (54). On basis of preclinical data and without prior exploratory phase 2 trial, a large international pivotal randomized phase 3 trial have been launched comparing the efficacy of cisplatin alone *versus* cisplatin-ombrabulin in 355 doxorubicin and ifosfamide-pretreated advanced soft tissue sarcoma patients (NCT00699517) (50). Noteworthy, cisplatin as single-agent is not regarded as active drug in sarcoma patients. There were some signs of activity: the objective response rate was 1% *versus* 4% and median PFS was slightly improved 1.4 *versus* 1.5 months (**Table 4**), however these figures could not be regarded as clinically meaningful. In absence of clinically relevant sign of activity in human malignancies, the development of this investigational drug had been stopped (55).

##### Aflibercept

Aflibercept is a recombinant fusion protein that traps circulating VEGF and PlGF (**Table 1**). Aflibercept alone have been assessed in a stratified non-randomized phase 2 trial in uterine sarcoma patients with 2 strata: leiomyosarcoma and so-called carcinosarcoma (data regarding carcinosarcoma are excluded from the present review, NCT00390234) (10) (**Table 2**). The study population is a mix of pre-treated and chemo-naïve patients. Overall, the results seem disappointing in both strata, without objective response, a time to progression inferior to 2 months. In the leiomyosarcoma cohort, it is difficult to interpret the PFR-6 since the study population is a mixed of both pre-treated and chemo-naïve patients.

##### Bevacizumab

Bevacizumab is a well-known monoclonal antibody that bocks VEGF-A (**Table 1**). The advantage of bevacizumab is that this antiangiogenic agent could be easily combined with classical chemotherapy. We gather the published trials assessing the activity of bevacizumab in sarcoma patients in 3 paragraphs: bevacizumab and vascular sarcomas (15-16), the gemcitabine/docetaxel/bevacizumab combination and bevacizumab in children sarcoma.

Agulnik et al. have assessed the activity of bevacizumab as single-agent in patients with angiosarcoma or epithelioid hemangioendothelioma (EHE) (NCT00288015; **Table 2**). Angiosarcoma are very aggressive sarcoma with very poor outcome. On the contrary, EHE are indolent malignancies that could be spontaneously stable for decades. The objective response rate was 2 (9%) of 23 in angiosarcoma patients, and the median PFS was somewhat disappointing: 3 months. In EHE patients, the objective response rate was 2 (29%) of 7. The reported median PFS and median OS were 9.8 and 35.5 months, respectively, reflecting the indolent natural course of the disease (15). Later the French Sarcoma Group, have launched a randomized phase II trial assessing the activity of weekly paclitaxel *versus* weekly paclitaxel plus bevacizumab in angiosarcoma patients (NCT01303497) (42) (**Table 3**). Grade 3 and 4 toxicities were more frequent in the combination arm: 44% *versus* 22%. Adding of bevacizumab did not improve the outcome (e.g., median PFS of 6 months with or without bevacizumab in this highly selected population). A similar US clinical trial has been closed for poor recruitment (NCT01055028). To conclude, bevacizumab did not appear particularly active in angiosarcoma, a paradigm of tumor angiogenesis.

The gemcitabine/docetaxel/bevacizumab combination has been assessed in three clinical settings: in pretreated soft tissue sarcoma patients (18), as first-line treatment in advanced soft tissue sarcoma (17, 19), and as neo-adjuvant treatment in soft tissue sarcoma patients (19), (**Table 1**). The randomized phase 3 trial (NCT01012297) conducted by Hensley et al. (51), clearly demonstrated that adding bevacizumab in 1^st^ line treatment did not improve the outcome (e.g., median PFS of 4.2 months with bevacizumab *versus* 6.2 months without bevacizumab; HR = 1.12 (95% CI: 0.74–1.70; p = 0.58) (**Table 4**).

Chilsholm et al. have reported the results of a large international randomized phase 2 trial assessing the added value of bevacizumab in chemo naive children with metastatic rhabdomyosarcoma (RMS) and non-RMS sarcoma (NCT00643565; **Table 3**) (43). This trial compared the chemotherapy combination (4 cycles of IVADo followed by 5 cycles of IVA) *versus* the same regimen with bevacizumab. Furthermore, non-progressing patients received 12 cycles of maintenance chemotherapy with cyclophosphamide/vinorelbine +/− bevacizumab according to the assigned arm. The statistical hypothesis was very ambitious: improvement of the median event free survival from 15.8 to 27.6 months (that represents a HR of 0.57). The objective response rate (ORR) was 36% versus 54% and the median OS was 20.5 *versus* 25.0 months. Nevertheless, regarding the primary endpoint that was event-free survival (EFS), authors observed a median EFS of 14.9 versus 20.6 months, but no statistical significance (HR, 0.93; 95% CI, 0.61−1.41; p = 0.72).The sample size did not allow analysis by histological subtypes. The rate of grades 3 and 4 adverse events of special interest (bleeding, thrombosis, congestive heart failure, wound healing complications) was similar in both arms (13%). The results of this trial are intriguing and may require confirmatory trial regarding the both clinically meaningful ORR and OS improvement.

To conclude, excluding metastatic RMS children patients, bevacizumab does not warrant further clinical investigation in sarcoma patients.

To conclude this section, we would like to discuss two studies very briefly.

First of all, the combination bevacizumab-doxorubicin has been assessed in a non-randomized 2-stage phase II trial, including seventeen patients with metastatic soft tissue sarcomas (16), (**Table 2**). Since the objective response rate was disappointing (12%), the accrual was stopped at the end of the 1st stage.

We would like also to mention in this review the following retrospective study assessing temozolomide and bevacizumab as treatment of advanced malignant solitary fibrous tumors (n = 14) (56). In this series, the partial response rate according to Choi criteria was 79% and the estimated progression-free survival was 9.7 months.

#### Tyrosine Kinase Inhibitors

##### Sunitinib

George et al. have reported a non-randomized phase 2 trial in 48 patients with advanced sarcomas (38) (**Table 2**). There was only one RECIST-based partial response in a desmoplastic round cell tumor patient. The PFR-6 was 7 (14.5%) of 48. Authors have noticed that significant decrease in SUV was seen in 10 of 21 patients assessable with FDG-PET. The data are difficult to interpret since the enrolled histological subtypes were heterogenous, evidence of disease progression at study entry was not required and the population included both chemo-naïve and pre-treated patients. Furthermore, sunitinib have been assessed as treatment of desmoid fibromatosis. The ORR was 26% (39) (**Table 2**). This result will be discussed later (*Sorafenib*). Of note, retrospective studies report the activity of sunitinib in extremely rare sub-types of sarcomas, such as solitary fibrous tumor or clear cell sarcomas (57, 58).

Three successive dose-escalating phase 1 trials have studied the association sunitinib with radiotherapy as neoadjuvant treatment for locally advanced but operable soft tissue sarcoma (59–61) (**Table 5**). The Lewin et al. trial has been prematurely closed for toxicity after enrollment of 9 patients (NCT00753727). Dose-limiting toxicities (DLT) were seen in four patients at first dose-level [50 mg per day for 2 weeks before radiotherapy, then 37.5 mg per day given during radiotherapy]. Despite dose-reduction, DLTs occurred in the two patients treated in dose-level -1 (Grade 3 hepatic cytolysis and Grade 3 neutropenia). There were one partial response and 8 stable diseases (59). Another dose-escalating phase trial had been reported (NCT01498835) (60). The 1st dose level was 25 mg per day starting 2 weeks before radiotherapy and dose-level 2 was 37.5 mg/day. There was only one DLT occurring in the 6 patients treated at the 1st dose-level (one grade 3 lymphopenia). There was no DLT occurring in the two patients treated at 2nd dose level. However, dose reduction of sunitinib was necessary in 5 of 9 patients. Tumor responses were partial response in one case, stable disease in seven cases and disease progression in one case. All patients underwent tumor resection (eight classified as R0). Pathological examination revealed ≥ 95% tumor necrosis in 3 of 9 resected specimens. In the third trial, a phase 2 trial, patients received sunitinib daily at the dose of 37.5 mg/day with a pre-operative radiotherapy of 45.0 to 50.4 Gy. Eight of 16 patients developed grade 3, and one patient developed grade 4, hematological toxicity. According to RECIST, there were three partial responses, 11 stable diseases and two disease progressions. Among the 16 patients, 14 underwent surgery. The proportion of tumor necrosis exceeded 90% in 5 of 14 patients, and four patients had postoperative complications requiring reintervention (61). Other clinical trials assess the same combination (NCT00753727; NCT01308034). To conclude, neoadjuvant treatment combining sunitinib and radiotherapy is associated with a significant toxicity; the clinical experience is limited and in absence of randomized trial, the added value of sunitinib could not be properly weighted.

**Table 5:** Trials assessing anti-angiogenic as neoadjuvant treatment.

Ref.	Pathology	Investigational treatment	n.	Clinical outcome	Pathological response
**Systemic treatments**					
**Navid et al. Int J Cancer 2017** (62)	Operable osteosarcoma	Bevacizumab, methotrexate, doxorubicin and cisplatin	31	4-year EFS = 57% 4-year OS = 83%	Good pathological response: 28%
**Ronellenfitsch et al. Ann Surg Oncol 2019** (63)	Localized soft tissue sarcoma	Pazopanib	21	No metabolic response	No patient with complete pathological response
**Munhoz et al. Oncologist 2015** (64)	Localized soft tissue sarcoma	Gemcitabine, docetaxel, pazopanib	5	No objective response	1 complete pathological response
**Verschraegen et al. Ann Oncol 2012** (19)	Localized soft tissue sarcoma	Bevacizumab + Gemcitabine + Docetaxel	15	ORR: 6/15 = 40% 2y-OS rate = 69%	2 complete pathological responses
**Concurrent radiotherapy and tyrosine kinase inhibitor**					
**Hass et al. Acta Oncologica 2015** (65)	Localized soft tissue sarcoma	Pazopanib	12	No objective response 0/12 DLT	Pathological response: 4/10
**Canter et al. Ann Surg Oncol 2014** (66)	Localized soft tissue sarcoma	Sorafenib	8	1 objective response 2/8 DLT	Pathological response : 3/8
**Meyer et al. CCR 2013** (67)	Localized soft tissue sarcoma	Sorafenib	18	- 3/18 DLT	Pathological response: 7/16
**Jakob et al. Radioth Oncol** (60)	Localized soft tissue sarcoma	Sunitinib	9	1 objective response 1/9 DLT	Pathological response: 3/9
**Lewin et al. BJC 2014** (59)	Localized soft tissue sarcoma	Sunitinib	9	1 objective response 4/9 DLT	–

EFS, event-free survival; OS, overall survival; ORR, objective response rate according to RECIST; DLT, dose-limiting toxicities.

##### Sorafenib

There are four trials assessing the activity of sorafenib as single-agent in pretreated soft tissue sarcoma (31–33, 35) (**Table 2**). The total number of enrolled patients in these 4 trials was 174 patients. Overall the reported ORR was 14 out of 149 assessable patients (9%). The PFR-6 widely differed according to the trial from 35% (35) to 0% (32). This difference in ORR reflects mainly the heterogeneity of study populations across the studies. Sorafenib was then assessed in different studies focusing particular diseases. The activity of sorafenib appeared somewhat disappointing in angiosarcoma patients (ORR of 14.6% and PFR-6 of 12%) (NCT00874874) (34) (**Table 2**).

In pretreated osteosarcoma, the reported ORR was only 3/35 (9%) and the PFR-6 was 17% (30) (EudraCT 2007-004396-19) (**Table 2**). Sorafenib and everolimus combination have been then assessed in advanced pretreated osteosarcoma in a single-arm trial, the ORR was 2/38 (5%) and the PFR-6 was 45% (17 patients) (NCT01804374) (68) (**Table 2**). Sorafenib was assessed in chordoma patients (NCT00874874). Chordoma are slowly growing tumors. The ORR was 2 out of 27 (7%) according to RECIST but 7 out of 27 (26%) according to Choi criteria. The PFR-6 was 85%, reflecting the relative indolent course of the disease (26) (**Table 2**). Then, sorafenib activity has been assessed in EHE patients. In this trial, evidence of disease progression at study entry was mandatory (NCT00874874). The ORR was 13% and the median PFS was 6 months (27) (**Table 2**).

The activity of sorafenib has been also assessed in desmoid fibromatosis patients with an expanded access program (37) (**Table 2**) and then a confirmatory placebo-controlled phase 3 trial (69). Desmoid fibromatosis is a very rare non-metastasizing infiltrative intermediate malignancy with unpredictable course, since spontaneous regressions could be seen (70). In the non-randomized trial, sorafenib at the dose of 400 mg/d provides an ORR of 6/24 (25%) according to RECIST; furthermore, decrease in MRI T2 signal intensity appeared as a metric of pharmacodynamic activity of sorafenib in this precise clinical setting (37). Later an investigator initiated confirmatory placebo-controlled phase 3 trial have been conducted in 87 patients with inoperable and with proven radiographic progression desmoid fibromatosis (NCT02066181) (69). The 2-year PFR was 81% compared to 36% (HR = 0.13; p<0.001). Before cross-over, the ORR was (16/49) 33% *versus* (7/35) 20%. There was one complete response with sorafenib. However, the tolerability of sorafenib could be discussed in the context of this non-life-threatening tumor; the occurrence of grade 3 toxicity was 29%, dose-reduction was needed in 65% of cases and 20% of patients discontinued sorafenib for toxicity (**Table 4**) (69). This trial stresses the fact that OR could be seen spontaneously in case of progressing fibromatosis desmoid.

There were 2 trials assessing combination in sarcoma patients with chemotherapy: sorafenib with dacarbazine (28) and sorafenib with ifosfamide (29) (**Table 2**). Both trials are non-randomized ones, both mixing pretreated and chemo naive patients. Evidence of disease progression at study entry was not mandatory in both trials. D’Amado et al. stressed that the ORR was 14% according to RECIST but 27.0% according to Choi criteria with sorafenib and dacarbazine combination (NCT00837148). The ifosfamide-sorafenib provides an ORR of 6/35 (17%) and the PFR-6 was 37%, however, most of patients were pretreated (NCT00541840). Both combinations appeared tolerable and active (since providing PFR-6 superior 14%), but we ignore if these figures are related to chemotherapy agent alone or are the consequence of a truly synergistic association.

Canter et al. have conducted a dose-escalating phase I trial assessing sorafenib and radiotherapy as neoadjuvant treatment of soft tissue sarcoma (NCT00864032). The reported objective response was 1 out 8 patients and the pathological response was 3 out of 8 patients. The association seemed tolerable (66).

To conclude, sorafenib is probably an active drug in osteosarcoma (30), chordoma (26), progressing EHE (27), but since there is no confirmatory randomized trial, the level of evidence remains low. On the contrary, since there is a convincing and well-conducted, sorafenib is for sure an active drug in desmoid fibromatosis; but regarding the tolerability, the optimal dose remains to be determined (69).

##### Tivozanib

To the best of our knowledge, there was only one trial assessing the activity of tivozanib in pretreated sarcoma patients. The figures suggest some activity: objective response rate of 3.4% and median PFS of 3.5 months. Nevertheless, PFR-3 and PFR-6 were not formally estimated (NCT01782313) (71) (**Table 2**).

##### Cediranib and Axitinib

Axitinib alone have been assessed in progressing patients with solitary fibrous tumor (SFT) in a non-randomized phase 2 trial (NCT02261207) (13). SFT are highly vascularized tumors. The authors measured an ORR of 11% according to RECIST but 78% according to Choi criteria. The median PFS was 9.4 months and the median OS was 25.3 months (**Table 2**). Wilky et al. have reported a phase I/II trial assessing the activity of the combination axitinib and pembrolizumab. There are preclinical data suggesting synergistic effects of anti-PD(L)-1 and anti-angiogenic agents. This synergistic effect had been clearly demonstrated in other clinical settings, especially in metastatic renal cell carcinoma. The axitinib and pembrolizumab combination provided an ORR of 10% in progressing soft tissue sarcomas (non-ASPS). The median PFS was only 3 months (NCT02636725) (14) (**Table 2**).

Both drugs have been assessed in ASPS patients. ASPS is a slowly growing tumor, with diffuse metastatic spreading including brain. ASPS are regarded as chemo-resistant. ASPS are particularly vascularized. Some case-reports suggest that ASPS are sensitive to immunotherapy (72). Cediranib had been assessed in one single-arm phase 2 trials (with 2 cohorts) and then in a randomized phase 2 trial. There was no objective response in 17 children patients at the dose of 12 mg/m^2^ (40). In the adult cohort, cediranib (30 mg/d) provided a response rate of 15/43 (35%) (NCT00942877); among these patients, 12 (26%) have been previously treated with another TKI (41) (**Table 2**). Regarding these conflicting results, a placebo-controlled randomized trial was welcome (44) (NCT01337401). In this trial, evidence of disease progression within 6 months before study entry was required. The primary endpoint was percentage change in sum of target marker lesion diameters between baseline and week 24. Median percentage change in sum of target marker lesion diameters for the evaluable population was −8.3% (IQR −26.5 to +5.9) with cediranib *versus* 13.4% (IQR +1.1 to +21.3) with placebo (p = 0.001). There was clinical meaningful improvement of outcome (**Table 3**). Lastly, the axitinib-pembrolizumab combination provided impressive results in ASPS patients: response rate of 54.5%, PFR-6 of 38% and median PFS of 12.4 months (14) (**Table 2**). To conclude, cediranib appears to be an active drug in progressing ASPS, but the development of cediranib is currently hold. The association TKI and immune check-point inhibitor appears promising in this clinical setting.

##### Anlotinib

Chi et al. have reported the results of a large stratified non-randomized phase 2 trials assessing the activity of anlotinib in advanced anthracycline-pretreated soft tissue sarcoma (NCT01878448). This drug is promising in all subgroups of patients providing a PFR-6 exceeding 14% (12) (**Table 2**). An international double-blind placebo-controlled phase 3 trial (NCT03016819) is ongoing in selected subtypes (ASPS, leiomyosarcoma and synovial sarcoma)

##### Pazopanib

Pazopanib is a multikinase inhibitor acting mainly on VEGF-R1, VEGF-R2 and VEGFR-3 (**Table 1**). The initial development of pazopanib in pretreated soft tissue sarcoma patients is perfect. There were 2 successive trials, first an exploratory phase 2 trial (23) and then a confirmatory phase 3 trial (NCT00753688) (52). Both have been conducted by the EORTC-STBSG. In the exploratory phase 2 trial, patients have been stratified according to histological subtypes; there were 4 subgroups: leiomyosarcoma, liposarcoma, synovial sarcoma and other sarcomas. In all cases, evidence of disease progression before study entry was mandatory. Excluding 2 cases, all patients have been chemotherapy pretreated. A Simon two-stage design have been applied for each histological sub-type (P1 = 40%; P0 = 20%; α = β = 0.1). The primary endpoint was PFR-3. The liposarcoma stratum have been closed after the 1^st^ stage due to lack of efficacy. Pazopanib appeared to be a promising drug in the 3 other subgroups: leiomyosarcoma (PFR-3 = 44%), synovial sarcoma (PFR-3 = 49%) and other sarcomas (PFR-3 = 39%) (**Table 2**). Later, a large international placebo-controlled phase 3 trial has been conducted to confirm the activity of pazopanib in non-adipocytic sarcoma patients. Cross-over was not allowed. The primary endpoint was PFS. Compared to placebo, pazopanib significantly improved the PFS (HR = 0.31, 95% CI 0.24–0.40; p<0.0001), but this did not translate into OS improvement (HR = 0.86, 95% CI 0.67–1.11; p = 0.25) (**Table 4**). Furthermore, this trial stressed that RECIST-based responses are rare with such drug: 6% in out of 246 patients treated with pazopanib (Van der Graaf et al. Lancet Oncol 2012). Based on this trial, pazopanib is approved in pre-treated non-adipocytic soft tissue sarcoma. Pazopanib was a first (and today the sole) TKI approved for management of no-GIST sarcoma patients.

Latter trials attempted to precise the activity of pazopanib in some rare histological subtypes. Pazopanib have been found active in ASPS (73) (NCT02113826), extra-skeletal myxoid chondrosarcoma (24) (NCT02066285), dedifferentiated SFT (20) (NCT02066285) (**Table 2**). Samuels et al. reported a second non-randomized phase 2 trial assessing the activity of pazopanib in progressing liposarcoma patients. The idea here is that liposarcoma is a heterogeneous group including dedifferentiated, myxoid/round cell, pleomorphic and mixed type liposarcomas. Grade 1 liposarcoma and well-dedifferentiated liposarcoma have been excluded. Overall, the ORR was low: 2%, the median PFS was 4.4 months, the PFR-3 was 68% and the PFR-6 was 39%. Dedifferentiated liposarcoma seemed relatively more sensitive to pazopanib, with PFR-3 of 74% and median PFS of 6.2 months (**Table 2**). Sadly, there is no confirmatory randomized trial assessing the activity of pazopanib in dedifferentiated liposarcoma (22).

Toulmonde et al. have reported a non-comparative randomized phase 2 trial assessing the activity of pazopanib in one hand and the activity of methotrexate-vinblastine in other hand in progressing desmoid fibromatosis (45) (NCT01876082) (**Table 3**). Cross-over was allowed. The ORR was 25% with chemotherapy and 37% with pazopanib. The PFR-6 was 45% and 84%, respectively. This trial results were in the line of previously reported one with sorafenib: multikinase inhibitors are active on progressive desmoid fibromatosis. Pazopanib warrants further clinical investigation in this clinical setting (45).

Subbiah et al. have reported a phase Ib/II trial assessing the activity of pazopanib-trametinib combination in sarcoma patients. The hypothesis is that MEK inhibitor, when inhibiting the oncogenic RAS/RAF pathway, could be able to overcome the resistance to VEGFR inhibitor. The primary endpoint was an uncommon one: PFR-4; the PFR-4 was 21%. Partial responses occurred in two patients (8%). With such figures it is difficult to conclude about the activity of the combination (25) (**Table 2**). Pautier et al. have reported recently the results of a large non-randomized phase 2 trial assessing the activity of gemcitabine-pazopanib in pretreated leiomyosarcoma (both soft tissue and uterine) (21) (NCT01442662). Gemcitabine is a largely used drug in leiomyosarcoma patients. The overall response rate according to RECIST was 23%, the median PFS was 6.5 months. But there were some concerns about the safety profile with Grade 3 to 4 neutropenia in 72% of cases, Grade 3 to 4 thrombocytopenia in 38%, Grade 3 to 4 hepatic cytolysis in 23%, and Grade 3 to 4 fatigue in 14% (**Table 2**). Regarding this safety profile, it is hard to believe that this combination could be used in everyday practice.

Pazopanib has been assessed as neoadjuvant treatment for locally advanced soft tissue sarcoma (63) (NCT01543802). As single agent, pazopanib failed to provide substantial activity, with only one metabolic response in 21 treated patients (63) (**Table 5**). Haas et al. have assessed the combination with radiotherapy in a phase I trial (65) (NCT01985295). There was no reported DLT. There was no objective response in 12 treated patients and 4 complete pathological response in 10 operated patients (65) (**Table 5**). These figures are in light that previously reported with other multikinase inhibitors.

To conclude, pazopanib is definitely an active drug in pretreated non-adipocytic soft tissue sarcoma. There is still a doubt about its activity in dedifferentiated liposarcoma.

##### Regorafenib

The initial development of regorafenib was initiated by academic groups. This strategy slightly differs from the earlier development of pazopanib. The strategy is based on exploratory placebo-controlled comparative phase 2 trials taken into account the histological subtypes. The idea here is to catch as soon as possible evidence for activity. The French Sarcoma and Austrian Groups has reported 2 successive comparative randomized placebo-controlled phase 2 trials assessing the activity of regorafenib in advanced pretreated sarcoma patients. Four parallel phase 2 trials have been conducted: liposarcoma, leiomyosarcoma, synovial sarcoma and other sarcomas. Regorafenib failed to improve PFS compared to placebo in liposarcoma patients: PFS was 1.1 months with regorafenib versus 1.7 months with placebo (HR 0.89 [95% CI 0.48–1.64] p = 0.70). These results are perfectly consistent with the trial assessing pazopanib. On the contrary, regorafenib significantly improved the PFS in pretreated leiomyosarcoma, synovial sarcoma and other sarcomas with respectively, HR = 0.46 [95% CI: 0.26–0.80] (p = 0.0045); HR = 0.10 [95% CI: 0.03–0.35] (p<0.0001) and HR = 0.46 [95% CI: 0.25–0.82] (p = 0.0061) (46) (NCT01900743) (**Table 3**). Later a fifth stratum in this randomized phase 2 trial have been launched in non-adipocytic soft tissue sarcoma both pretreated by anthracycline and pazopanib. There was also a significant improvement of PFS (median: 2.1 months with regorafenib *versus* 1.1 months with placebo) (adjusted HR = 0.33; 95% CI: 0.15–0.74; p = 0.007) and a large and nearly significant OS benefit despite the cross-over (adjusted HR = 0.49; 95% CI: 0.23–1.06; p = 0.07; median OS = 17.8 *versus* 8.2 months) (47) (**Table 3**). The activity of regorafenib is still currently under investigation, according to the same design: parallel placebo-controlled randomized phase 2 trials, in pretreated osteosarcoma, chondrosarcoma, Ewing sarcoma, and chordoma. The results of the osteosarcoma are fully published. The PFS with regorafenib was 4.1 months and the PFS was 1.0 with placebo. Despite cross-over, OS was 11.3 months with regorafenib and 5.9 in patients allocated to placebo arm (48) (NCT02389244) (**Table 3**). In a comparative phase 2 trial in osteosarcoma, median PFS was 1.7 months with placebo compared to 3.6 months (HR= 0.42; 95% CI, 0.21–0.85; p = 0.017) (49) (NCT02048371) (**Table 3**). Other ongoing trials are ongoing in rhabdomyosarcoma, liposarcoma, Ewing sarcoma and angiosarcoma (NCT02048371; NCT02048722). To conclude, regorafenib is an active drug in pretreated advanced non-adipocytic soft tissue sarcoma and osteosarcoma. In both trials, there was a trend in OS improvement despite cross-over. These results are particularly encouraging since regorafenib seems active after pazopanib exposure.

### Focus on Some Histological Subtypes

#### Angiosarcomas

Angiosarcoma is an aggressive malignancy with very poor outcome at metastatic stage. Doxorubicin, paclitaxel, gemcitabine provide short-term responses. The assessment of activity of anti-angiogenic agents in advanced angiosarcoma makes sense, since the phenotype of this sarcoma and the frequent occurrence of KDR mutations (74), involving in the angiogenic signaling pathways. Nevertheless, results of clinical trials did not demonstrate high sensitivity of angiosarcoma to anti-angiogenic agents. Bevacizumab alone provides a response rate of 9% with a median PFS of 3 months (15). Adding of bevacizumab did not improve the efficacy of weekly paclitaxel (42). Sorafenib provides a median PFS of 1.8 months in cases of superficial angiosarcoma and 3.8 months in visceral angiosarcoma (34). In few words, available evidence does not demonstrate that angiosarcoma are particularly sensitive to anti-angiogenic.

#### ASPS

ASPS is one of the rarest sarcomas. ASPS are usually diagnosed in adolescents and young adults. ASPS could spread to lung and brain. This relapse can occur several decades after initial management, requiring long-term follow-up. At diffuse metastatic stage, watchful follow-up is an acceptable option because of the spontaneous indolent course of the disease (at metastatic stage, 5-year overall survival often exceeds 90% in large case series). ASPS is regarded as refractory to classical chemotherapy. ASPS is characterized by the recurrent unbalanced translocation der(17)t(X;17)(p11;q25) and its 2 mutually exclusive variants of inherent chimeric protein fusion (ASPSCR1-TFE3), that induce an immunosuppressive micro-environment, an overexpression of MET and angiogenesis *via* HIF1α overexpression (14). Cediranib provides a response rate of 19% and 35% in 2 trials (41, 44). Pazopanib provides a response rate of 1 out 6, with a median PFS of 5.5 months (73). The most impressive results have been obtained with the combination axitinib-pembrolizumab: the response rate was 6 out of 11, with a median PFS of 12.4 months (14). Combination of immune check-point inhibitor and anti-angiogenic tyrosine kinase inhibitor requires further clinical investigation and international collaborative efforts regarding the extreme rarity of this histological subtype.

#### Desmoid Fibromatosis

Desmoid fibromatosis are low-grade conjunctive tissue tumors without metastatic potential. Watchful follow-up is now the upfront management of this disease, since only one third on desmoid fibromatosis will progress. In case of disease progression, there is no consensus on treatment. We have used different treatment with very limited level of evidence for activity (hormonal therapy, chemotherapy, imatinib …). Two recent trials have now demonstrated that antiangiogenic agents are clearly active in management of desmoid fibromatosis. Gounder et al. have conducted a superiority phase 3 trial demonstrating that sorafenib (400 mg/day) significantly improve the PFS compared to placebo in progressing desmoid fibromatosis. This trial stressed also that placebo could induce objective response in 20% of cases (69). A second trial shows promising activity of pazopanib (25). Multikinase inhibitors constitute a breakthrough in management of desmoid fibromatosis. Nevertheless, the optimal dose that tackles disease progression without inacceptable toxicity is an open question for this non-malignant disease.

#### Liposarcomas

Pazopanib and regorafenib are both obviously inactive on liposarcoma. Multikinase inhibitors could act on sarcoma through angiogenesis inhibition as well as by direct anti-proliferative effect. The intrinsic mechanism of resistance of liposarcoma to multikinase inhibitors have to be identified. The tumor enrichment in cancer stem cells could be a mechanism of resistance that needs further preclinical exploration (75). Liposarcoma is a heterogeneous group of tumors. There is still a doubt about activity of multikinase inhibitors in advanced dedifferentiated liposarcoma, ideally this requires a dedicated placebo-controlled randomized trial.

### Focus on Clinical Settings

#### Neoadjuvant Setting 

The aims of neoadjuvant are here: (i) obtain tumor shrinkage to perform less morbid surgery or lead to surgery initially not operable tumor (ii) obtain massive destruction of tumor cells, and (iii) in case of systemic treatment eliminate micro-metastasis and reduce the risk of metastatic relapse. Main results in the neoadjuvant setting are presented in **Table 5**.

Ronellenfitsch et al. have recently reported a single-arm phase II trial assessing pazopanib alone as neoadjuvant treatment for soft tissue sarcoma. There was only one FDG-PET metabolic response out 21 enrolled patients and only one completed pathological response. These findings are regarded as disappointing (63).

Different combinations of multikinase inhibitors with radiotherapy have been assessed as neo-adjuvant in locally advanced soft tissue sarcoma. The published trials are exploratory ones (dose-seeking phase I and non-randomized phase II trials). The toxicity profile requires lower dose of multikinase inhibitors compared to the dose used as single-agent. Overall, the tumor shrinkage according to RECIST is rare with three reported partial response out of 56 enrolled patients (5%). Complete pathological response or pathological response superior to 90% of tumor surface was reported in 17/35 cases (49%). The limited follow-up did not allow assessment of metastatic relapse after local management (23–25, 42, 62).

Combination of chemotherapy and multikinase inhibitors have been assessed as neoadjuvant treatment for soft tissue sarcoma and osteosarcoma. Navid et al. have reported the results of a single-arm phase II trial assessing methotrexate, doxorubicin, cisplatin, and bevacizumab as preoperative treatment of osteosarcoma. The rate of good pathological response was 28%, the 4-year EFS and 4-year OS were 57% and 83%, respectively. These figures are within the range of results obtained with classical chemotherapy alone (62). Verschraegen et al. have assessed bevacizumab, gemcitabine and docetaxel as neoadjuvant treatment in 15 patients with soft tissue sarcoma; the overall response rate was 40%) (19). Munhoz et al. have reported a dose-escalating phase I assessing the tolerability of the combination of pazopanib, gemcitabine and docetaxel in soft tissue sarcoma. The trial had been prematurely closed after treatment of five patients since there was one progressive disease and four stable disease according to RECIST. Out of 4 operated patients, there was complete pathological response in one patient. Toxicity was significant, especially asthenia (64).

At the end, in absence of randomize trial, it is very challenging to measure the real impact of adding multikinase inhibitor to neoadjuvant radiotherapy or chemotherapy. Nevertheless, the preliminary results are not encouraging.

#### Adjuvant Setting

To the best of our knowledge, there is no published trial exploring anti-angiogenic as adjuvant treatment in sarcoma.

#### Advanced Disease

To the best of our knowledge, we have found only 2 published non-randomized trials assessing the role of antiangiogenic agent in chemo-naïve soft tissue sarcoma patients. Both trials assessed the activity of the combination Bevacizumab, Gemcitabine, Docetaxel (17, 19). Verschraegen et al. have assessed the activity of this combination as 1^st^-line treatment in 20 advanced soft tissue sarcoma patients. The best response rate was 25%, the median PFS was 5.0 months. The median overall survival was 11.0 months (19). Dickson et al. have assessed the activity of this combination in 35 patients with advanced soft tissue sarcoma. Among these 35 patients, 29 of them were chemo-naïve (83%). The ORR was 49%. The PFR-3 and PFR-6 were 76% and 65%, respectively. These figures suggest that this combination is active and warrants further clinical exploration. Nevertheless, gemcitabine + docetaxel combination has been formally compared to classical 1^st^-line treatment with doxorubicin in the GeDDIS trial (76). The combination did not improve the outcome and the safety profile favored doxorubicin.

Most of trials focused on pre-treated patients. Regarding the data available, we can conclude that pazopanib is active in non-adipocytic soft tissue sarcoma, regorafenib is also active in non-adipocytic soft tissue sarcoma pretreated by chemotherapy but also pretreated by pazopanib. Regorafenib provides promising sign of activity in pretreated osteosarcoma. Lastly, sorafenib is active in progressing desmoid fibromatosis. All these evidences came from randomized trials.

### Imaging

#### Choi Criteria

RECIST remains the most largely metric used for drug development. Excluding the intriguing case of desmoid fibromatosis, there is no doubt that tumor shrinkage is the sign of drug activity and not the consequence of the natural course of the disease. Nevertheless, anti-angiogenic agents induce different change in tumor tissue, especially necrosis or cavitation. “Choi” criteria largely used in GIST management could be also applied to soft tissue sarcoma treated by anti-angiogenic. According to these criteria, partial response is defined by decrease in tumor size ≥10% or decrease in tumor density ≥15% on CT-scan. The decrease in tumor density catch the treatment-induced necrosis. These criteria could be more appropriate to assess the activity of anti-angiogenic in sarcoma patients. For example, Martin-Broto et al. have reported the activity of pazopanib in advanced dedifferentiated SFT. According to RECIST, there were only two partial responses out of 35 patients (6%). According to Choi criteria, there were 18 partial responses out of 35 patients (51%). Choi criteria were better than RECIST criteria for identifying patients with worse overall survival; median OS of patients with progressive disease according to Choi was 4.5 months whereas median OS of patients with progressive disease according to RECIST was 6.5 months (20). Choi criteria should be considered as useful tool in anti-angiogenic development in sarcoma patients.

#### Tumor Cavitation

Tumor cavitation is a pattern of response, but this could lead to complications, e.g. tumor rupture of peritoneal or abdominal mass with hemorrhage or peritonitis or pneumothorax. In the PALETTE trial, the reported incidence of pneumothorax was 8/246 (3%). Pneumothorax could be bilateral that is a life-threatening condition. Risk factors for pneumothorax have been analyzed in two retrospective studies. Sabath et al. have identified pre-treatment cavitation (odds ratio [OR] = 7.0, p<0.001) and pleural or base location of lung nodules (OR = 10.4, p<0.001) as independent risk factors (77). Nakano et al. have identified diameter of 30 mm or more (OR = 13.3, p = 0.039) and prior history of pneumothorax (OR = 16.6, p = 0.045) as independent risk factors (78). To the best of our knowledge, there are no validated criteria defining response according to the occurrence of cavitation in tumor masses.

#### Metabolic Response

FDG-PET could be in theory used to monitor the pharmaco-dynamic effects of such drugs. For example, Ronellenfitsch et al. have assessed the activity of pazopanib as neoadjuvant treatment in soft tissue sarcoma patients with FDG-PET. Objective response was defined as >50% reduction of the mean standardized uptake value (SUVmean) in post-treatment compared to pretreatment FDG-PET. Mean change in SUVmean of post- *versus* pretreatment PET was a 6% decrease (range: - 65% to +34%). There was only one objective response. Nevertheless, among the 21 enrolled patients, 15 were liposarcoma patients. The one patient with metabolic response had grade 2 undifferentiated sarcoma of the lower leg. The tumor showed 70% regression with hyaline necrosis and did not reduce in size during neoadjuvant therapy. Among the 17 operated patients, there is no correlation between SUV change and pathological response (63).

### Health Quality of Life

In the PALETTE trial, quality of life has been extensively assessed in pretreated non-adipocytic sarcoma treated with pazopanib compared to those treated with placebo. Overall, this study provides three major information: (i) self-reported symptoms scales catch the toxicity profile of pazopanib, (ii) but, health Quality of life was not alter by pazopanib treatment, and (iii) there is an improvement of PFS without impairment of health quality of life (79). In the placebo-controlled phase 2 REGOSARC trial assessing regorafenib compared to placebo, Berry et al. have used a different method: Quality-adjusted time without symptom and disease progression (Q-TWIST). The Q-TWIST was 8.0 months with regorafenib compared to 5.7 months with placebo (p<0.0001) (80). Overall, despite their toxicity profiles, multikinase inhibitors seem to provide meaningful results of health quality of life. We strongly recommend to integrating such analysis in further randomized trials.

### Predictive Factors

Since PALETTE trial met the primary objective, this is the appropriate frame to explore predictive factors for clinical benefit of multikinase inhibitor in sarcoma patients. Sleijfer et al. have reported extensive analysis of correlation between clinical benefit and circulating biomarkers. For example, levels of PlGF and VEGFR2 at 12 weeks were both correlated with overall survival (81). At the end, there is no obvious and convincing correlation between change in circulating biomarkers and clinical benefit. Some clinical parameters were associated with long-term survival: good performance status, low tumor grade and normal hemoglobin level. Nevertheless, these parameters are of limited importance to guide the decision making in daily practice. To conclude, in sarcoma patients (as well as in other clinical setting – such as renal cell carcinoma), there is no validated predictive factors that could identify patients with high probability of response to multikinase inhibitors (82).

### Pharmacological Consideration

The use of oral mutikinase inhibitor expose to drug-drug interaction. This had been markedly stressed by Mir et al. in case of pazopanib. The absorption of pazopanib requires low pH at gastric level. The use of anti-acid significantly alters the PFS and the OS of sarcoma patients treated with pazopanib (83). The impact of other drug-drug interactions needs to be analyzed in further clinical studies, including real-life studies. The optimal dose of TKI is an open question. For example, in the REGOSARC trial about 2/3 of patients treated with regorafenib have required dose-reduction for tolerability issues. In the trial assessing the activity of sorafenib in desmoid fibromatosis, the chosen dose was 400 mg per day rather than 600 mg as used in other malignancies. The circulating concentration of sorafenib is rarely done in everyday practice, but we could monitor it and adjust at individual level the dosage of TKI in each patient. Overview of dose-escalating phase I trial assessing TKI demonstrates that objectives responses and long lasting stable disease could be seen with low dose of TKI (84). It is of major importance since the role of palliative systemic treatment is to alleviate symptoms and improve quality of life.

## Discussion and Unresolved Questions

The main limitation of this systematic review is that most of published trials are non-randomized ones, regarding the heterogeneity in sarcomas, the interpretation of data coming from such non-randomized trials (e.g. identification of meaningful sign of activity) remains challenging. Overall, macro-molecules acting as anti-antiangiogenic agents (bevacizumab, angiotensin, aflibercept, ombrabulin) provide disappointing results in clinical trials. On the contrary, mutikinase inhibitors (especially pazopanib and regorafenib) that act as anti-angiogenic agents and also as anti-proliferative agents constitute a breakthrough in advanced pretreated soft tissue sarcoma and advanced pretreated osteosarcoma. Both drugs are inactive in liposarcoma; this intrinsic resistance of liposarcoma needs to be better understood. Regorafenib remains active after exposure to pazopanib. Regorafenib development is ongoing in other clinical settings: chondrosarcoma, chordoma, rhabdomyosarcoma … There are still unresolved questions: the ideal dose of TKI, the ideal imaging method to monitor activity, the identification of relevant drug-drug interaction, the identification of predictive biomarkers. Development of TKI in sarcoma is a good example of win-win partnership between pharmaceutical companies and academic researchers. Nevertheless, because most of trials are not randomized and enrolled limited and very heterogeneous population, no definitive conclusion could be drawn; an doubts remain on the true activity of some TKI on some particular subtypes of sarcoma (especially the rarest ones and bone sarcoma).

## Author Contributions

Conception of the review: NP. Data collection: NP, LL, P-YC. Quality control of data: LL, P-YC. Data analysis and interpretation: NP, LL. Drafting the article: NP. Critical revision of the article: NP, LL, TR, P-YC. Final approval of the version to be published: NP, LL, TR, P-YC. All authors contributed to the article and approved the submitted version.

## Conflict of Interest

The authors declare that the research was conducted in the absence of any commercial or financial relationships that could be construed as a potential conflict of interest.

The reviewer GG declared a past co-authorship with one of the authors NP to the handling editor.

## Abbreviations

95% CI, 95% confidence interval; ASPS, alveolar soft part sarcoma; c-Kit, stem cell factor receptor; CT-scan, computed tomography scanner; DLT, dose-limiting toxicities; EFS, event-free survival; EHE, epithelioid hemangioendothelioma; EORTC-STBSG, European Organisation for Research and Treatment of Cancer - Soft Tissue and Bone Sarcoma Group; FDG-PET, ^18^F-fluorodeoxyglucose (FDG) positron emission tomography (PET); GIST, gastrointestinal stromal tumor; HR, hazard ratio; IC50, Inhibitory concentration 50; IQR, interquartile range; IVA, ifosfamide vincristine actinomycin; IVADo, Ifosfamide Vincristine Actinomycin Doxorubicin; KDR, kinase insert domain receptor; MEK, mitogen-activated protein kinase; MRI T2, magnetic resonance imaging T2 time; OR, odds ratio; ORR, objective response rate; OS, overall survival; PD1, programmed death 1; PDGFR-α, platelet-derived growth factor receptor alpha; PD-L1, programmed death-ligand 1; PFR, progression-free survival rates; PFR-3, progression-free survival rate at 3 months; PFR-6, progression-free survival rate at 6 months; PFS, progression-free survival; PIGF, placental growth factor; Q-TWIST, quality-adjusted time without symptom and disease progression; RECIST, response evaluation criteria in solid tumors; RMS, rhabdomyosarcoma; SFT, solitary fibrous tumor; SUV, standard uptake value; TKI, tyrosine kinase inhibitors; VEGF, vascular endothelial growth factor; VEGF-R, vascular endothelial growth factor-receptor.
